# Resistive Switching Characteristics Improved by Visible-Light Irradiation in a Cs_2_AgBiBr_6_-Based Memory Device

**DOI:** 10.3390/nano11061361

**Published:** 2021-05-21

**Authors:** Fengzhen Lv, Tingting Zhong, Yongfu Qin, Haijun Qin, Wenfeng Wang, Fuchi Liu, Wenjie Kong

**Affiliations:** 1 Guangxi Key Laboratory of Nuclear Physics and Technology, School of Physical Science and Technology, Guangxi Normal University, Guilin 541004, China; zhongtingting0101@163.com (T.Z.); qinyongfu168168@163.com (Y.Q.); qinhaijun2020@163.com (H.Q.); wkong@mailbox.gxnu.edu.cn (W.K.); 2 Ningbo Institute of Materials Technology and Engineering, Chinese Academy of Sciences, Ningbo 315201, China; wangwf@nimte.ac.cn

**Keywords:** light modulation, Cs_2_AgBiBr_6_, bipolar resistive switching behavior, bromine vacancy, space-charge-limited current mechanism, Schottky-like barrier

## Abstract

Light-modulated lead-free perovskites-based memristors, combining photoresponse and memory, are promising as multifunctional devices. In this work, lead-free double perovskite Cs_2_AgBiBr_6_ films with dense surfaces and uniform grains were prepared by the low-temperature sol-gel method on indium tin oxide (ITO) substrates. A memory device based on a lead-free double perovskite Cs_2_AgBiBr_6_ film, Pt/Cs_2_AgBiBr_6_/ITO/glass, presents obvious bipolar resistive switching behavior. The *R*_OFF_/*R*_ON_ ratio under 445 nm wavelength light illumination is ~100 times greater than that in darkness. A long retention capability (>2400 s) and cycle-to-cycle consistency (>500 times) were observed in this device under light illumination. The resistive switching behavior is primarily attributed to the trap-controlled space-charge-limited current mechanism caused by bromine vacancies in the Cs_2_AgBiBr_6_ medium layer. Light modulates resistive states by regulating the condition of photo-generated carriers and changing the Schottky-like barrier of the Pt/Cs_2_AgBiBr_6_ interface under bias voltage sweeping.

## 1. Introduction

Since the first physical demonstration based on memristor theoretical concepts proposed by L. O. Chua, resistive random access memory (RRAM) based on resistive switching (RS) characteristics has been extensively explored for novel data storage [1,2]. So far, RS characteristics are typically regulated by an electric field. However, to further improve stability, reduce consumption, increase density, and enhance versatility, multi-field coordinated regulation has attracted widespread attention in RRAM [3,4,5,6,7]. Therein, electrically/optically controlled RS behavior has the potential to build multifunctional optoelectronic memristor devices, simplify the complexity of programmable logic circuits, and reduce costs [6,8,9]. Furthermore, a suitable light signal can ensure a large memory window and multiple storage levels of RRAM [9,10]. Among the various photoelectric materials, perovskites have attracted attention due to their physical properties at the beginning of the studies of optoelectronic memristors. Therein, metal oxide perovskites, e.g., BiFeO_3_, SrRuO_3_, SrTiO_3_ and so on, have demonstrated to possess excellent photoelectric memristor characteristics [11,12,13]. However, the high-temperature fabrication process limits the industrial production of ceramic perovskites. Lead halide perovskites (APbX_3_, with A = Cs^+^, CH_3_NH_3_^+^, (H_2_N)_2_CH^+^, etc.; X = I^−^, Br^−^, or Cl^−^) have attracted attention in RRAM due to their bandgap tunability, ambipolar charge transport, and long charge diffusion length [6,14,15,16]. However, the intrinsic thermal instability of MA and the toxicity of soluble Pb limit the eventual commercialization of lead halide perovskite–based RRAM [17,18]. In order to overcome the above disadvantages, lead-free alternatives, a new generation of double perovskites with a formula of A_2_M^+^M^3+^X_6_ (A or M^+^ = monovalent cation, M^3+^ = trivalent cation, X = halide anion) have recently generated widespread research interest [19,20,21]. Therein, silver-bismuth double perovskites (e.g., Cs_2_AgBiBr_6_) have become one of the most important materials in solar cells, photodetectors, and photovoltaic devices due to their higher efficiencies and suitable bandgaps comparable to that of other members, such as Cs_2_AgInBr_6_, Cs_2_InSbCl_6,_ and Rb_2_AgInBr_6_ [19,20,22]. The RS characteristics of silver-bismuth double perovskites have been observed recently, whereas the light regulation of RS characteristics has not been extensively researched.

In this study, we prepared high-quality Cs_2_AgBiBr_6_ (CABB) films on indium tin oxide (ITO)–coated glass substrates using a sol-gel method. Bipolar resistive switching (BRS) behavior was observed in the Pt/CABB/ITO/glass devices. The stability of the RS characteristics and ON/OFF ratio was enhanced by light regulation at a wavelength of 445 nm. An RS mechanism based on energy band bending and carrier transportation was proposed and discussed. Electrical conduction analysis indicated that the RS behaviors of CABB films are primarily attributed to the trap-controlled space-charge-limited current (SCLC) conduction caused by Br vacancies (V_Brs_) in the CABB layers. Light regulates the RS behavior of Pt/CABB/ITO/glass devices by changing the height or width of the Schottky-like barrier in the Pt/CABB interface under applied bias voltages. The excellent photoelectric storage performance of CABB-based memory devices in this work provides preferable choices for the commercialization of photoelectric memory.

## 2. Materials and Methods

### 2.1. Spin-Coating Process for CABB Film Growth

ITO/glass substrates were cleaned by sequential sonication in acetone, isopropyl alcohol, and deionized water (20 min each). After drying nitrogen, the precursor solution was prepared by combining cesium bromide (CsBr, 99.5%), silver bromide (AgBr, 99.9%), and bismuth bromide (BiBr_3_, ≥98%) at a molar ratio of 2:1:1 with anhydrous dimethyl sulfoxide (DMSO, ≥99.8%). The yellowish solution was stirred for 12 h at 65 °C in an Ar-filled glove box. The above reaction solution was dripped onto ITO/glass substrates and sequentially spin-coated at 500 rpm for 15 s and 5000 rpm for 45 s. Finally, the target samples were obtained by annealing on a hot plate at 280 °C for 300 s.

### 2.2. Characterization

The X-ray diffractometry (XRD) patterns of the CABB films were investigated using an XRD system (MiniFlex600, Rigaku Corporation, Tokyo, Japan). The morphology of the produced sample was characterized by field emission scanning electron microscopy (SEM; Helios G4 CX, Zeiss Auriga Inc., Oberkochen, Germany). The ultraviolet-visible (UV–Vis) absorption spectrum was measured on a Shimadzu spectrophotometer (UV-2700, Shimadzu Corporation, Kyoto, Japan). The conduction state of CABB-based devices under applied bias voltages was examined by conducting atomic force microscopy (CAFM; Bruker Dimension Icon Inc., karlsruhe, Germany). The elemental composition was analyzed via X-ray photoelectron spectroscopy (XPS; ESCALAB250Xi, Thermo Fisher Scientific Inc., Waltham, MA, USA) using Al K_α_ radiation. Ultraviolet photoelectron spectroscopy (UPS; Nexsa^TM^, Thermo Fisher Scientific Inc., Waltham, MA, USA) was performed to examine the band structure of Pt/CABB/ITO/glass device. The ST-500-(4TX-6PORTS) micro manipulated probe system (Janis Research Company, LLC., Woburn, MA, USA), which combines a Keithley 2400 SourceMeter and a laser source with the 445 nm wavelength and 0~100 mW output power (BF61064, Changchun New Industries Optoelectronics Technology Co., Ltd., Changchun, China), was applied to measure the RS characteristics of the Pt/CABB/ITO/glass devices in air at room temperature. The laser beam irradiated the devices through a 200 mm fiber reserved in the probe system during the measured processes.

## 3. Results and Discussion

**Figure 1:**
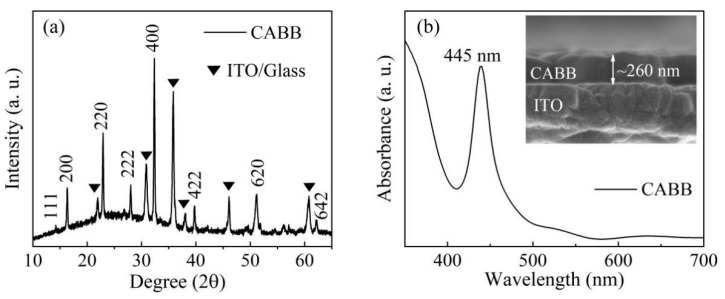
Basic physical characteristics of CABB films. (**a**) XRD patterns of the cubic CABB film; (**b**) optical absorption spectrum of the CABB film. The inset displays the cross-sectional SEM image of CABB layers spin-coated on the ITO/glass substrate.

As shown in Figure 1a, XRD peaks are distributed in the (111), (200), (220), (222), (400), (422), (620), and (642) planes of crystalline CABB, suggesting the formation of a cubic perovskite structure [22]. The UV–Vis absorption spectrum was characterized at room temperature. As depicted in Figure 1b, an obvious absorption band was distributed in the region from 410 to 500 nm, and a sharp absorption peak was located at 445 nm. A cross–sectional SEM image of the CABB/ITO/glass heterojunction presents a CABB layer with a uniform thickness of ~260 nm, as shown in the inset of Figure 1b.

**Figure 2:**
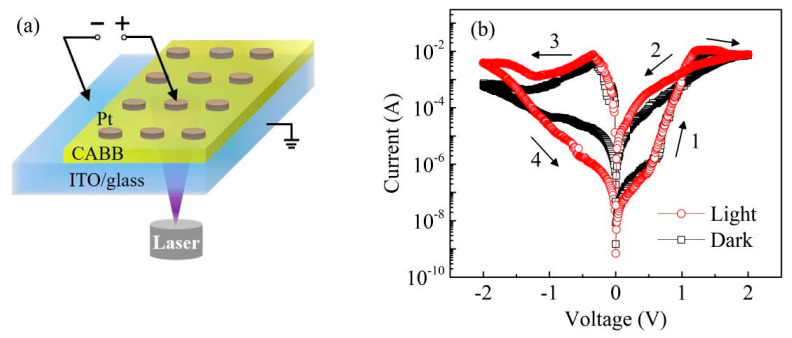
(**a**) Schematic of the Pt/CABB/ITO/glass devices used for RS performance measurements. (**b**) The semi-logarithmic plot of *I*–*V* curves of the CABB-based device in the dark and under light illumination (445 nm, 4.67 mW/cm^2^) with a sweep voltage of 2 V. Arrows and numbers represent the sweeping direction and sequence of bias voltages, respectively.

Figure 2a exhibits the device configuration and diagram of the Pt/CABB/ITO/glass multilayer structure. A ~100 nm thick Pt layer patterned by a metal mask with a diameter of 300 µm was sputtered on the CABB/ITO/glass heterojunction as the top electrode (TE). DC voltages in a sequence of 0 V⟶ 2V⟶ −2 V⟶ 0 V were applied on the Pt electrode of the device, and the compliance current (*I*_cc_) was 0.1 A. *I*–*V* curves were measured to explore the resistance changes in dark and light-illuminated environments (wavelength of 445 nm, 4.67 mW/cm^2^). As shown in Figure 2b, the Pt/CABB/ITO/glass device possesses typical BRS characteristics under bias voltage sweeping. The device switches from the high-resistance state (HRS) to the low-resistance state (LRS) at a set voltage of +2 V (*V*_SET_) and from LRS to HRS at a reset voltage of −2 V (*V*_RESET_). The current values in LRS under violet-light irradiation are higher than that in the dark, namely, the BRS behavior becomes more obvious under light illumination.

**Figure 3:**
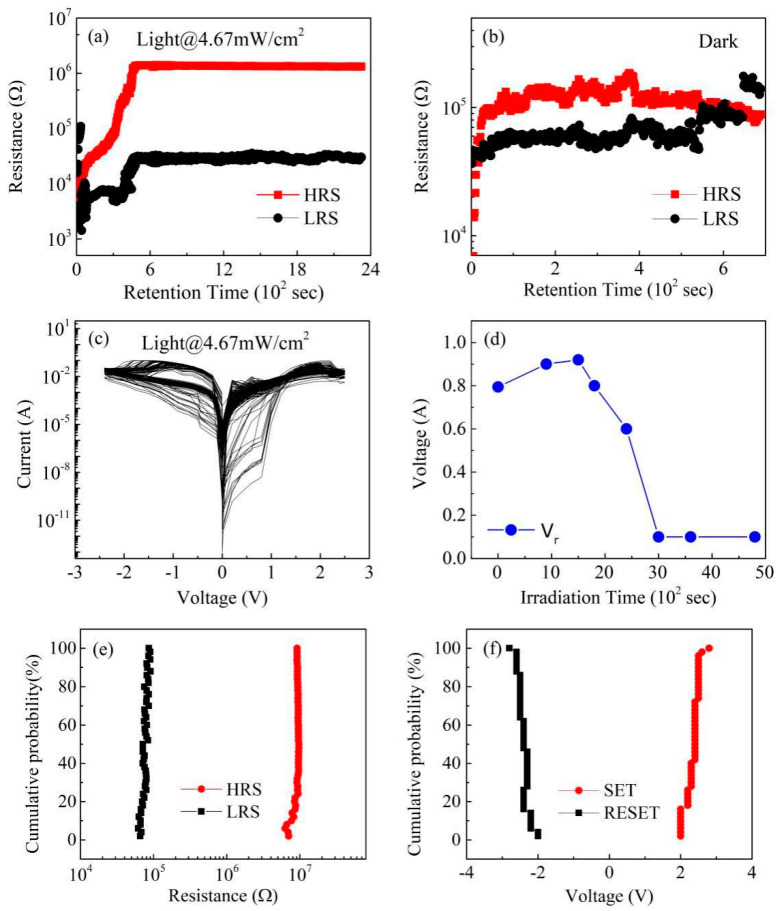
Resistance vs. time measured at 0.1 V after poling by +2 and −2 V under violet–light irradiation (**a**) and in dark conditions (**b**). (**c**) *I*–*V* sweeps of the Pt/CABB/ITO/glass device for 500 cycles under light illumination. (**d**) The relationship of *V*_r_ and exposure time tested under the light irradiation of 445 nm wavelength. (**e**) Cumulative resistance distribution of HRS and LRS extracted from 100 cycles in the Pt/CABB/ITO/glass device. (**f**) The distributions of the SET and RESET voltages.

To evaluate the effects of light illumination on RS storage ability, the retention ability was measured under electric pulses of *V*_SET_ and *V*_RESET_ in light and dark conditions, respectively. As shown in Figure 3a, LRS (i.e., ON state) and HRS (i.e., OFF state) were maintained for over 2400 s at a reading voltage (*V*_r_) of 0.2 V under light, and the ratio of the OFF state and ON was about 2 × 10^2^. However, the *R*_OFF_/*R*_ON_ ratio in dark conditions was less than that under illumination, and the LRS failed to HRS over 450 s, indicating that the retention of the device was weakened (Figure 3b). Thus, the retention characteristics can be obviously enhanced under light irradiation modulation. We surmised that the LRS retention failure was caused by the diffusion of vacancies existing in the CABB layer. As previously reported, the retention time (*t*_c_) is related to a thermal activation behavior, which obeys Arrhenius equation as follows [23,24]:

$$t_{c}={\left(\frac{N_{0}}{N^{*}}\right)}^{2}\frac{1}{\pi D_{0}}e^{\left(\frac{E_{\alpha }}{kT}\right)}$$

where *N*_0_ is the total number of vacancies scaled over the device area, *D_0_* is the diffusion coefficient of vacancies, N* is the critical density at the center of the vacancy-filament, *E_a_* is the activation energy of vacancies, *k* is Boltzmann’s constant, and *T* is the thermal temperature. Under the electric field, the vacancies migrated and formed the conductive filament (CF) due to their low activation energy in the RS layer. However, the vacancies concentration inside the filament gradually reduced with the vacancies inside conductive filaments (CF) diffusing spontaneously. When the concentration reduced below a critical value, the LRS retention failed. Figure 3c presents that the BRS characteristics of Pt/CABB/ITO/glass devices can be sustained about 300 cycles under bias sweeping. With the irradiation time increasing, the value of *V*_r_ was required to read the same ratio of HRS and LRS decreased, as shown in Figure 3d. Figure 3e exhibits the distribution of HRS and LRS measured at *V*_r_ during 100 switching cycles in the Pt/CABB/ITO/glass device. The coefficient of variation (CV) values of HRS and LRS are 10.0% and 9.1%, respectively, indicating that the resistance distributions are fine uniform. When the device was repeatedly switched between HRS and LRS, *V*_SET_ and *V*_RESET_ distributed in a narrow range of 2 V to 2.6 V and −2 V to −2.8 V, respectively. The CV values are 7.8% and 6.4%, indicating that the device maintains uniformity for the reset and set voltages (Figure 3f). Above all, light irradiation can effectively improve RS storage characteristics in CABB–based memory devices.

**Figure 4:**
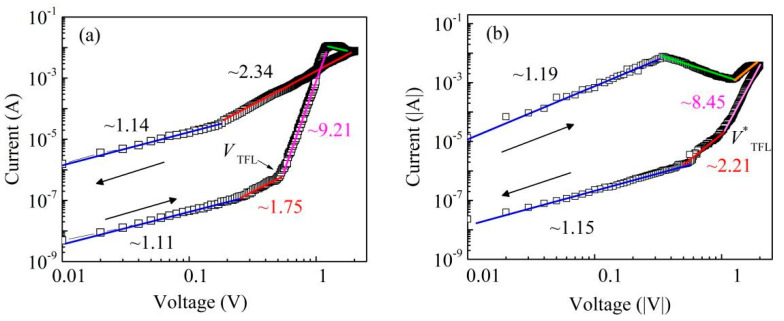
Log-log plot of *I*–*V* for (**a**) the positive bias region (0~2 V) and (**b**) the negative bias region (0 to −2 V) under light illumination.

Presently proposed RS mechanisms include CF model, space-charge-limited current (SCLC), Schottky emission, and Poole-Frenkel emission. These mechanisms can be distinguished using an isothermal logarithmic plot of the *I*–*V* curves. Figure 4 shows the double logarithmic plots of the *I*–*V* curves for the positive and negative bias regions in Pt/CABB/ITO/glass devices. As shown in Figure 4a, when a low positive voltage (0~0.3 V) is applied to a Pt electrode, the fitting slope of the log(*I*)–log(*V*) curve is 1.11, i.e., the relationship of *I* and *V* follows Ohm’s law (*I*∝*V*) and the device maintains the HRS. With increasing positive voltage, the fitting slopes of the lines are about 1.75 and 9.21, respectively, which indicates that the carrier transport behavior is dominated by SCLC (*I*∝*V*^2^ and *I*∝*V*^n^, n > 2) [21,25,26]. The transition between the ohmic and SCLC behavior indicates trap distribution in the CABB films. With the positive voltage further increasing, the current sharply dropped in the higher voltage region (1.2–1.8 V), which signified an apparent decrease in conductivity, which is the footprint of negative differential resistance (NDR) [27]. Then, when the positive voltage arrives at *V*_SET_, the device switches from the HRS to the LRS. Similarly, the negative region shows the same general trend as the positive region (Figure 4b). In order to investigate the type of traps, XPS was applied to analyze the variation in valence states.

**Figure 5:**
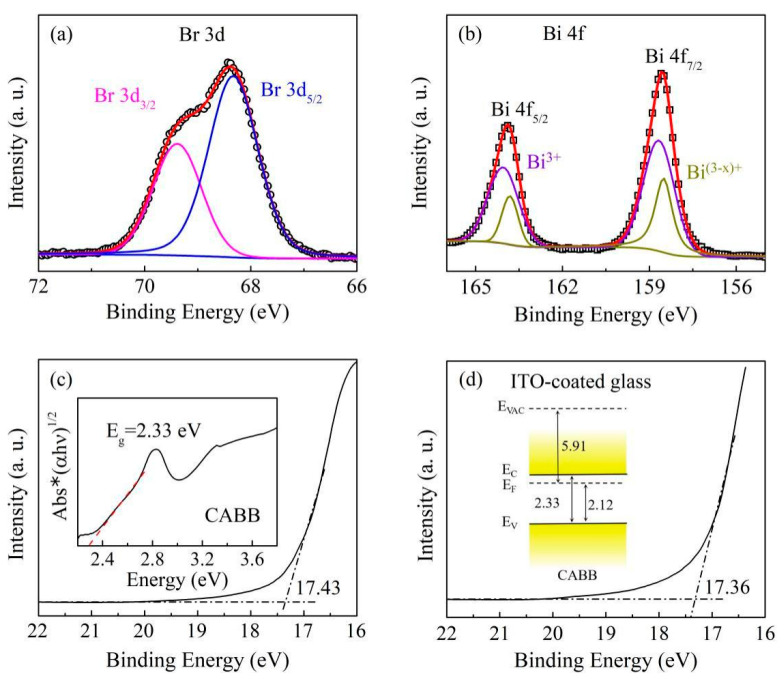
The core-level spectra of (**a**) Br 3d and (**b**) Bi 4f with peak fittings. (**c**) The UPS spectrum of the CABB film at the cut-off region measured by the He I source (*hν* = 21.22 eV). The inset is the Tauc plot obtained from UV–Vis spectroscopy. (**d**) The cut-off region of the ITO-coated glass. The inset is a schematically illustrated band diagram of the CABB film.

As shown in Figure 5a, the Br 3d peak is composed of a doublet with 3d_3/2_ and 3d_5/2_ signals at 69.38 and 68.33 eV. The Br 3d peaks of the CABB films slightly towards the positive, which indicated that abundant V_Brs_ exists in the CABB film [28]. Figure 5b shows the Bi 4f core-level spectrum. The main peaks at 164.85 and 159.47 eV are attributed to Bi^3+^, and two peaks with lower binding energies at 163.81 and 158.50 eV are ascribed to the low valence state Bi^(3−x)+^, also suggesting the formation of V_Brs_ [29]. In addition, to investigate the conditions of injected charge carriers and intrinsic surface defects in the conductive process, UPS was conducted to examine and clarify the contact types of the Pt/CABB/ITO/glass devices. As shown in Figure 5c, the work function of the CABB film was calculated to be 5.91 eV, which is similar to the value in previous reports [30,31]. This is close to the value of the ITO/glass substrate, which is 5.86 eV, as shown in Figure 5d. Therefore, the contact between the CABB layer and ITO–coated glass is ohmic, and a Schottky contact forms at the interface of Pt/CABB due to the different work functions of Pt (~6.4 eV) [32,33]. Combined with the result of the Tauc plot obtained from the UV–Vis spectra of the CABB film, the band gap of CABB film was determined to be 2.33 eV and the valence band (VB) maxima as 2.12 eV. This indicates that the Fermi level is located near the conduction band (CB), which shows that a larger number of hole-trap states are distributed above the VB [34,35].

**Figure 6:**
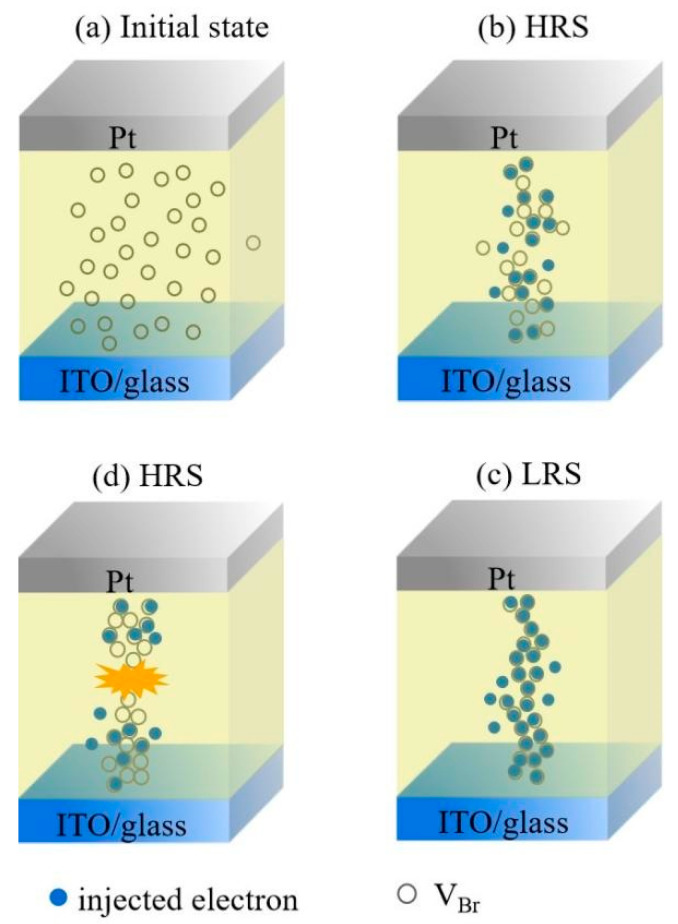
Schematic illustration of the conductive mechanism for the operation of the Pt/CABB/ITO/glass device. (**a**) Initially, the device is in the HRS due to the random distribution of V_Brs_. (**b**) V_Brs_ captures injected electrons and moves toward ITO under a positive bias. (**c**) The conductive filament of V_Brs_ forms in the CABB layer and switches the device to LRS. (**d**) The filament is broken and the device switches into HRS.

**Figure 7:**
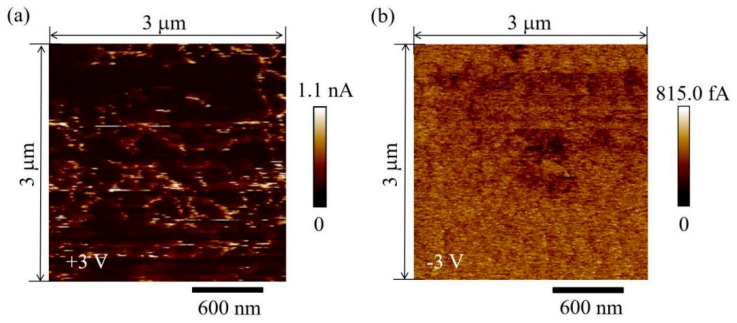
CAFM images of the ON state under (**a**) +3 V bias voltage and OFF state under (**b**) −3 V bias voltage. All image sizes are 3 μm × 3 μm.

Figure 6 displays the conductive process in the Pt/CABB/ITO/glass device. Initially, V_Brs_ randomly dispersed in the CABB layer. When a positive bias was applied on the Pt electrode, the injected electrons concentration was lower than the concentration of the thermally generated free carriers in the CABB layer; the *I-V* relationship follows Ohm’s law. The device did not conduct electricity and stayed at HRS in the low positive bias region (0 < V < 0.3 V) (Figure 6a). As the forward voltage increased (0.3 V < V < 0.5 V), the electrons injected from ITO were captured by V_Brs_ in the CABB layer, and the *I-V* relationship obeyed Child’s law (Figure 6b). As the forward bias further increased and arrived at the trap-filled limit voltage (*V*_TFL_, 0.5 V), the trapped electrons were activated and released from the trap centers, and additional injected electrons could not immediately fill in these traps due to their high activation energy [36]. Typically, the traps were always filled, and this conductive behavior switched to trap-free SCL conduction and obeyed *I*∝*V*^n^. The above process is known as the trapping process in the SCLC mechanism. As the forward voltage reaches *V*_SET_, the injected electrons cannot fill the trap but the trap maintains a filled-state; the electron concentration is high, and the device switches to the LRS. Furthermore, the rich regions of V_Brs_ expand toward the ITO cathode and eventually forms CF due to their lowest activation energy, and the injected electrons can migrate by vacancy hopping [37]. Thus, the device stays at the LRS (Figure 6c). The device can remain in the LRS even though the bias voltage sweeps in reverse. When the bias crosses *V*_RESET_ and reaches V*_TFL_ (−1.0 V), the trapped electrons are drawn out from the trap centers, the current behavior recovers SCL conduction, and the *I-V* relationship recovers to *I*∝*V*^2^. This process is also termed detrapping. Meanwhile, the filaments of V_Brs_ were ruptured as a result of the Br ion motion being influenced by negative voltage, and the spontaneous diffusion of V_Brs_. Thus, the device switches from LRS to HRS (Figure 6d). The formation and rupture of V_Brs_ were confirmed by CAFM measurements under different voltages (+3 and −3 V). As shown in Figure 7, conductive channels appear in the ON state (+3 V) and disappear in the OFF state (−3 V). When the Pt/CABB/ITO/glass device is exposed to light conditions, the photo-generated hole (h^+^) can recombine with Br^−^, Br^−^ + h^+^⟶Br, causing more V_Brs_ accumulation in the CABB layer, where more conductive channels composed of V_Brs_ participate in the RS under bias voltage sweeping [6]. As shown in Figure 2b, the current under illumination is higher than that in dark conditions.

**Figure 8:**
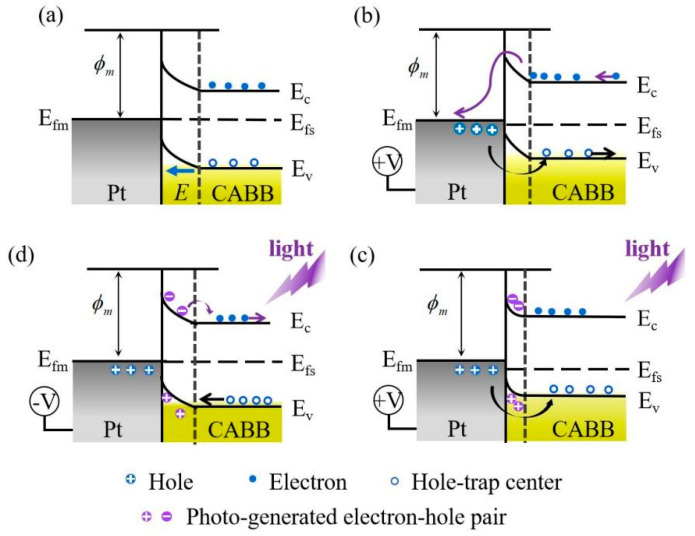
Photo-assisted switching mechanism in the Pt/CABB/ITO/glass device. (**a**) Initial HRS: a Schottky-like barrier generated in the Pt/CABB interface prevents electrons transport between Pt and ITO; (**b**) SET process: vacancies located at the CABB surface are filled, the height and width of the barrier are decreased; (**c**) photo-assisted SET process: holes left by photo-generated electrons intensify the band bending and facilitate electron transport across the thinner barrier in the Pt/CABB interface; (**d**) RESET process: photo-generated electrons move toward the ITO and holes recombine with the trapped electrons in the depletion region, the barrier recovers its original state.

According to previous reports, light irradiation principally regulates the RS behavior by modulating the Schottky-like barrier in the photoelectric memory [10,34,38]. Thus, we propose that the barrier reversibly changes with bias voltage sweeping upon light illumination. Figure 8a depicts a schematic of a band diagram for the initial state (V = 0) of the Pt/CABB junction in dark conditions. Initially, some of the electrons in the CB of the CABB layer move spontaneously toward the TE Pt owing to the lower Fermi level of Pt, while leaving behind trapped holes to form the space charge region near the Pt/CABB interface. Thus, the energy band of the CABB film bends upward to form a Schottky-like barrier and generate a built-in electrical field that points from CABB the Pt/CABB interface. They prevent electrons transferring from the CABB layer to the TE Pt, and the Pt/CABB/ITO/glass device remains in HRS. When the Pt electrode is positively biased and increases to *V*_SET_ in the dark, the external electric field weakens the internal electrical field and the height of the Schottky-like barrier is lowered. V_Brs_ in the depletion region, which is near the Pt/CABB interface, is gradually filled with electrons injected from the BE ITO, leading the width of the Schottky-like barrier is narrowed (Figure 8b). Additionally, the holes can be injected from the Pt electrode across the Pt/CABB interface and fill the interfacial hole-trap centers, leading to a lowered barrier [34]. Thus, the Pt/CABB/ITO/glass device switches from HRS to LRS. When the device is exposed to optical illumination with a wavelength of 445 nm (~2.79 eV), electrons located at the VB of the CABB jump into the CB after absorbing photons and become free-moving charges, namely, photo-generated electrons. They leave behind photo-generated h_s_^+^ in the VB to intensify the band bending at the Pt/CABB interfacial region and constantly facilitate electron transport across the junction with a thinner barrier (Figure 8c). Continuous illumination hinders the recombination of electron-hole pairs and further reduces the inhibiting effect of the barrier. Therefore, the current of the device examined under light illumination is higher than that in dark conditions, as shown in Figure 2b. When the TE Pt is negatively biased (*V*_RESET_) under 445 nm illumination, the photo-generated electrons move toward the BE ITO, whereas photo-generated h_s_^+^ move toward the Pt/CABB interface to recombine with the electrons trapped in the depletion region. Additionally, some trapped electrons in V_Brs_ can also be photo-excited to the CB and the V_Brs_ move toward the Pt/CABB interface. Then, the captured holes slowly drift back to their original state. The barrier recovers its original height and width, and the device switches back to its original HRS (Figure 8d).

## 4. Conclusions

To summarize, obvious bipolar RS characteristics were observed in a Cs_2_AgBiBr_6_ film prepared by a sol-gel-assisted spin coating method. The RS behavior was principally attributed to the trap-controlled SCLC mechanism, and charge traps composed of bromine vacancies were considered to play a key role in forming the conductive paths. Optical illumination with a laser of 445 nm and 4.67 mW/cm^2^ energy density effectively modulated the RS behavior and enhanced the stability of the Pt/Cs_2_AgBiBr_6_/ITO/glass devices. A higher ON/OFF ratio of ~200 and a longer data retention of over 2400 s were measured in light conditions. A possible trap-mediated RS mechanism based on the existence of hole trapping centers at the Pt/Cs_2_AgBiBr_6_ interface was proposed to explain the illumination modulation. This work can benefit the design and application of optoelectronic memory devices based on lead-free double perovskites.

## Data Availability

All data generated and analyzed during this study are included in this article and the attached supporting information.
